# Toward a More Efficient Implementation of Antifibrillation Pacing

**DOI:** 10.1371/journal.pone.0158239

**Published:** 2016-07-08

**Authors:** Dan Wilson, Jeff Moehlis

**Affiliations:** Department of Mechanical Engineering, University of California Santa Barbara, Santa Barbara, CA 93106, United States of America; Gent University, BELGIUM

## Abstract

We devise a methodology to determine an optimal pattern of inputs to synchronize firing patterns of cardiac cells which only requires the ability to measure action potential durations in individual cells. In numerical bidomain simulations, the resulting synchronizing inputs are shown to terminate spiral waves with a higher probability than comparable inputs that do not synchronize the cells as strongly. These results suggest that designing stimuli which promote synchronization in cardiac tissue could improve the success rate of defibrillation, and point towards novel strategies for optimizing antifibrillation pacing.

## Introduction

While cardiac arrest continues to be a leading cause of death in the industrialized world, for the past century, the only clinically reliable method of defibrillation has been the application of a high voltage shock across the myocardium, typically with a voltage gradient of at least 5V/cm [1]. Patients who survive an initial cardiac arrest are at a higher risk for subsequent cardiac arrests and often require implantable cardioverter defibrillators (ICDs) in order to improve long term survival rates [2, 3]. This chronic treatment is not without side effects, however, as defibrillating shocks from ICDs cause intense pain, which puts patients at a severe risk of secondary effects including depression and anxiety [4–7], adversely affecting their quality of life. Furthermore, these shocks can lead to long term damage, including fibrosis [8], as well as other short-term side-effects [9]. This has led researchers to search for new, low-energy defibrillating stimuli in order to reduce pain and other side effects associated with these defibrillating shocks. While researchers have investigated possibilities such as optimizing stimulus waveforms [10] or determining shocks of minimum intensity to achieve defibrillation [11], neither of these strategies yield stimuli which can defibrillate painlessly. Others have developed pacing strategies which can terminate single spiral waves responsible for tachycardia [12] [13], but these strategies are not effective when multiple spirals are present, as is the case during cardiac arrest.

Recently, a new class of defibrillation strategies has emerged [14–17], which attempts to eliminate spiral waves responsible for cardiac fibrillation with a series of low energy electric shocks. It has been shown that in the presence of an electric field, anisotropy in conductivity between cardiac myocites can result in local depolarization or hyperpolarization of the cells [18–20]. This anisotropy in the tissue can be caused by fiber curvature, [21], gap junction discontinuities, [22], or the presence of intracellular clefts [23] such as blood vessels or fatty tissue. If the local depolarization becomes large enough, this tissue anisotropy can create “virtual electrodes” or “secondary sources” from which spreading waves of depolarization can emanate. It has been proposed that these virtual electrodes are responsible for progressively synchronizing the myocardial tissue over the course of multiple shocks, at which point fibrillation is eliminated. It was shown in [14] that as long as the shock frequency is higher than the dominant frequency of spiral wave oscillations, the virtual electrodes will be able to perturb the spiral wave filament, and synchronize the surrounding tissue. However, no framework currently exists for optimizing the pulse timing.

It has been suggested that synchronization of myocardial activity is important for preventing reentry of spiral waves after a defibrillating shock [24–26], and in this work, we propose a methodology for experimentally determining an efficacious sequence of pulses to synchronize the activity of the myocardial cells, increasing the likelihood of eliminating reentrant spiral waves. This strategy only requires the ability to measure action potential durations (APDs) and Diastolic Intervals (DIs) which could be done experimentally. Furthermore, this methodology could readily handle multiple types of stimuli (e.g. strictly hyperpolarizing) if they were available in an experimental setting. Using this newly developed methodology, we are able to determine pulsing patterns which defibrillate at lower energies than pulsing patterns with a fixed frequency.

## Bidomain Equation Simulation

We consider a bidomain model for cardiac simulations. The governing equations for the intracellular (*V*_*i*_) and extracellular (*V*_*e*_) potential are [27, 28]

$$\begin{array}{ccc} \nabla \cdot {\bar{\sigma }}_{i}\nabla V_{i} & = & \beta I_{m},\\ \nabla \cdot {\bar{\sigma }}_{e}\nabla V_{e} & = & -\beta I_{m},\\ I_{m} & = & C_{m}\frac{\partial V_{m}}{\partial t}+I_{\text{ion}}-I_{\text{stim}},\\ V_{m} & = & V_{i}-V_{e}. \end{array}$$ (1)

**Fig 1:**
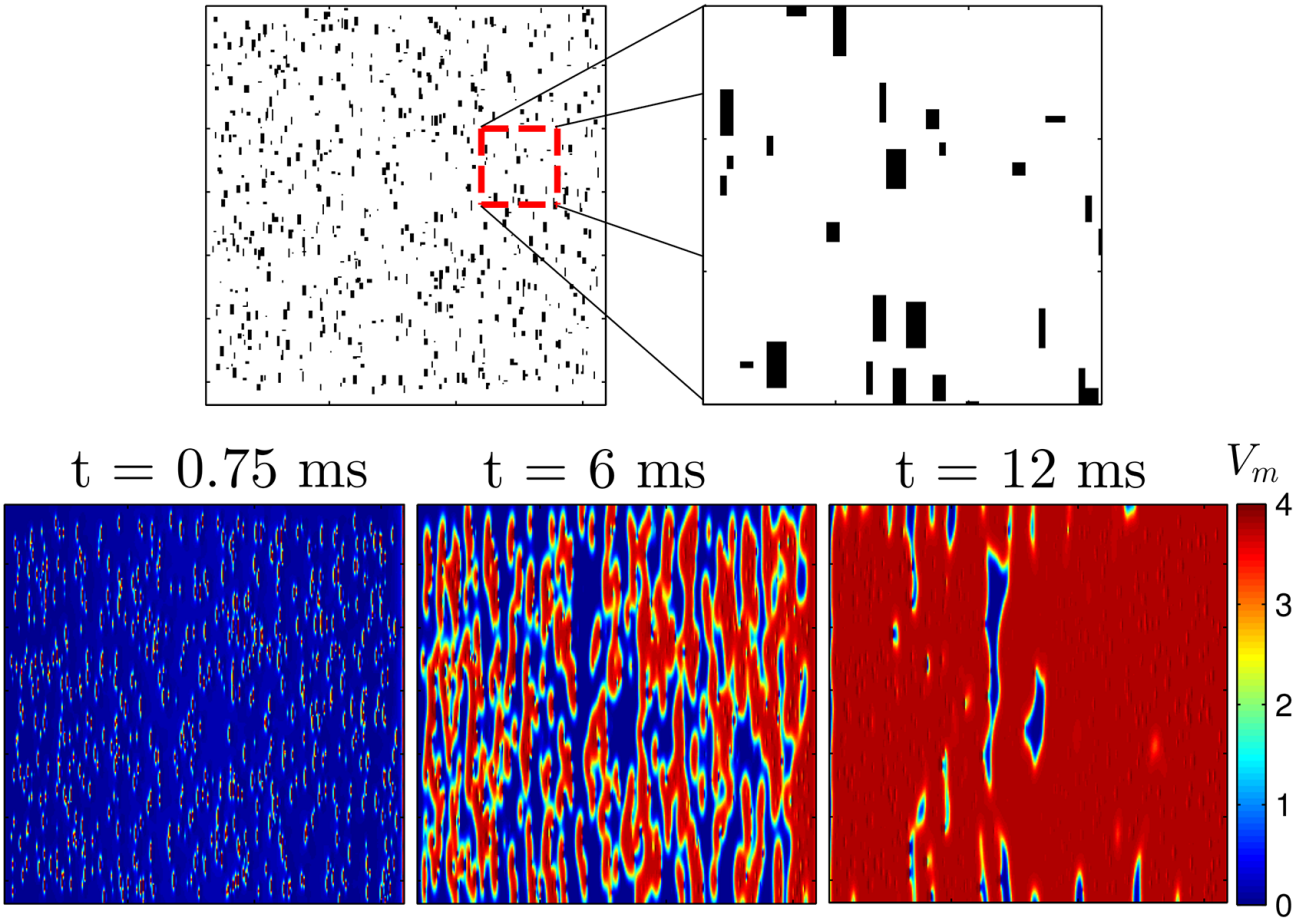
The top panels show an example of randomly chosen sets of gap junctions removed in Eq (1), shown in black. In the bottom panels a voltage gradient of 1500 units applied from left to right to a 2D sheet of quiescent cells Eq (2) for a duration of 1.5 ms. Because of the removal of the gap junctions, virtual electrodes start to form soon after the voltage gradient is applied. By 12 ms, nearly all cells in the domain have been excited. The colorbar presented here applies to all simulations using the Karma model.

Here, ${\bar{\sigma }}_{i}$ and ${\bar{\sigma }}_{e}$ intra- and extracellular conductivity tensors, respectively, *β* is the surface to volume ratio of the membrane, *C*_*m*_ gives the cell capacitance, *V*_*m*_ is the transmembrane voltage, and *I*_stim_ represents an external current density, which might come from an external pacemaker. *I*_ion_ gives the cell’s ionic current density, which is determined from the individual cell dynamics. To make the following analysis more concrete, we begin by using the Karma model for cardiac activity [29]:

$$\begin{array}{ccc} I_{\text{ion}}\left(V_{m},n\right) & = & -\tau_{V}^{-1}f\left(V_{m},n\right),\\ \dot{n} & = & \tau_{n}^{-1}g\left(V_{m},n\right), \end{array}$$ (2)

where *n* represents a gating variable. For an explanation of all constants and functions, we refer the reader to [29], with parameters Re = 1.34 and *M* = 4 chosen so that spiral wave breakup is observed in tissue. In bidomain simulations, we take the intracellular and extracellular domain to be a square. An extracardiac space extends past the cardiac tissue on the sides which are transverse to the principal fiber direction (on the left and right sides of the domain in Fig 1). In these simulations the extracardiac space represents two percent of the overall domain. The extracardiac potential, *V*_*o*_, obeys $\nabla \cdot {\bar{\sigma }}_{o}\nabla V_{o}$, where ${\bar{\sigma }}_{o}$ is the extracardiac conductivity tensor. Along the boundary of the cardiac tissue and the extracardiac space, the following conditions hold

$$\begin{array}{ccc} {\bar{\sigma }}_{i}\nabla V_{i}\cdot \eta & = & 0,\\ V_{e} & = & V_{o},\\ {\bar{\sigma }}_{e}\nabla V_{e}\cdot \eta & = & {\bar{\sigma }}_{o}\nabla V_{o}\cdot \eta . \end{array}$$ (3)

Here, *η* represents a vector normal to the boundary. We assume that an electrode mandates the value of *V*_*o*_ on the sides of the extracardiac space transverse to the principal fiber direction. On the remaining boundaries of the domain, we impose no flux boundary conditions. In realistic hearts, the conductivity is anisotropic, and in these two-dimensional simulations we take

$$\begin{array}{c} {\bar{\sigma }}_{i}=[\begin{array}{cc} g_{ix}\left(x,y\right) & 0\\ 0 & g_{iy}\left(x,y\right) \end{array}],\;{\bar{\sigma }}_{e}=[\begin{array}{cc} g_{ex}\left(x,y\right) & 0\\ 0 & g_{ey}\left(x,y\right) \end{array}],\;{\bar{\sigma }}_{o}=[\begin{array}{cc} g_{ox}\left(x,y\right) & 0\\ 0 & g_{oy}\left(x,y\right) \end{array}]. \end{array}$$ (4)

Furthermore, in these simulations, we model the insulating plaque [30–32] that can form in older hearts, by setting *g*_*ix*_ = 0 in some areas (which represents the removal groups of gap junctions that are perpendicular to the principal fiber direction (c.f. [14, 16]).

Defibrillating pulses are modeled by applying an external voltage gradient from left to right in the bottom panels of Fig 1, transverse to the principal fiber direction. The effect of defibrillating shocks will produce a complicated pattern of depolarization and hyperpolarization near the virtual electrodes, but the main effect of the virtual electrodes is to produce wavefronts of excitation in quiescent cells which quickly excite surrounding quiescent tissue. For this reason, for the remainder of this manuscript, we will refer to this type of stimulation as an excitatory pulse. In order to formulate a computationally tractable control objective, we will approximate each virtual electrode as a point source of excitation for refractory tissue, i.e. we assume that the effects induced by small perturbations to each cell caused by the virtual electrodes (c.f. [33]) are negligable so that they only provide a means of eliciting action potentials. Also, we assume that during a defibrillating pulse, the virtual electrodes will depolarize tissue in an all-or-nothing fashion, meaning that increasing or decreasing the applied voltage can only serve to increase or decrease the size and overall number of virtual electrodes, respectively, based on the distribution of size and shape of conductivity discontinuities present in the myocardial tissue.

## Dynamic Programming to Determine a Pulsing Pattern

**Fig 2:**
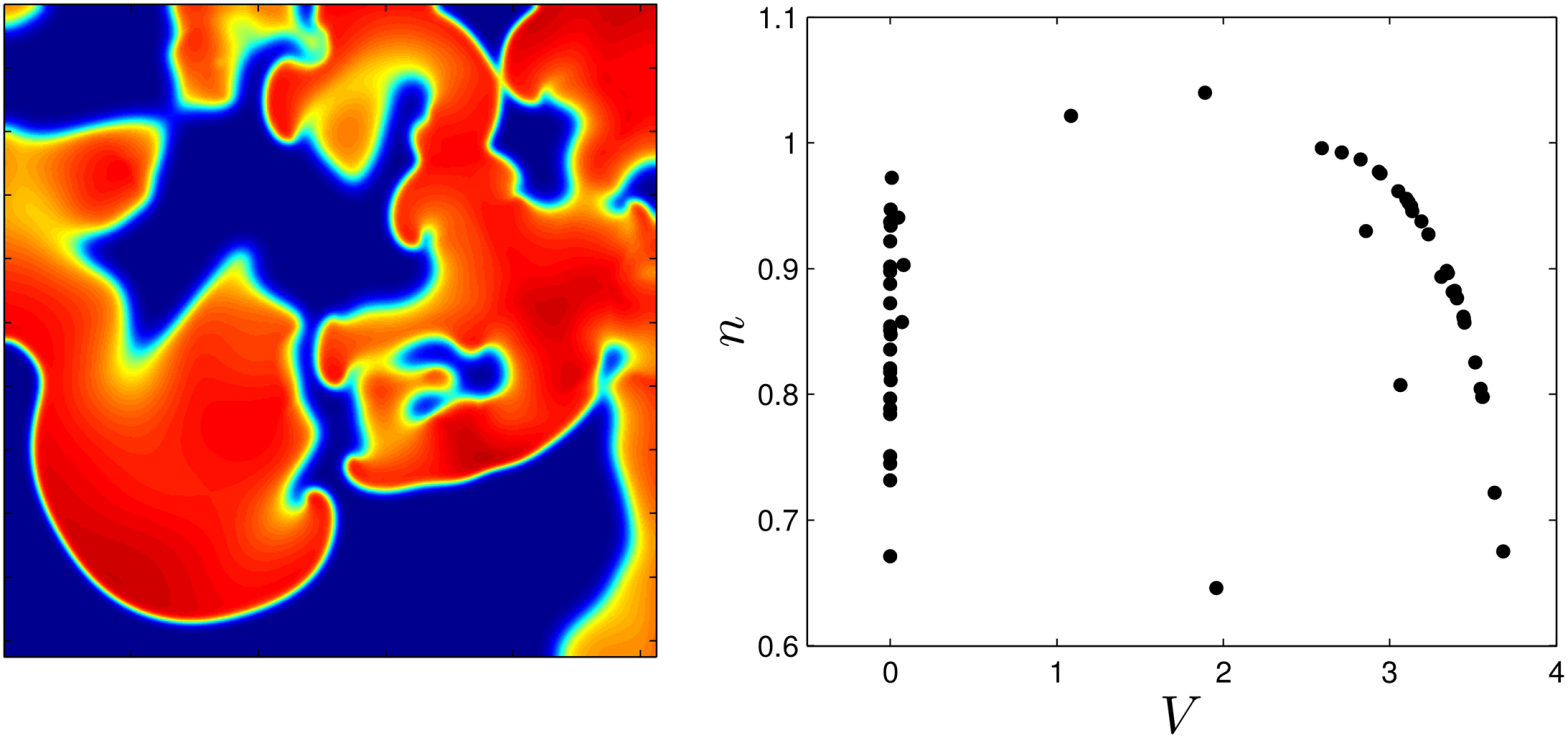
The left panel shows the medium with many spiral wave cores present. The right panel shows the states of 250 cells, chosen to provide a uniform sampling over the grid.

We initiate a spiral wave in the medium and as time progresses, the initial spiral breaks into multiple spirals. The left panel of Fig 2 shows multiple spiral waves within the medium, and the right panel shows the states of some of the individual cells, evenly distributed throughout the medium. We find that during defibrillation, most cells remain close to a transient attractor [34, 35], which in this example is a one-dimensional manifold which the cells tend to follow on their approach to the fixed point. Each cell follows the transient attractor towards the fixed point until it is reexcited by the next wave front.

Similar to the work in [36], our goal is to synchronize the activity of the cells within the myocardium in order to terminate spiral activity, however here we use a different method. We will assume that the defibrillating stimulus acts on a time scale much shorter than the natural period of the spiral waves so that cells are predominantly reexcited either directly by the virtual electrodes or by the wave fronts created by the virtual electrodes, (see Fig 1), but that very few cells will be excited by reentrant spiral waves. We formulate a dynamic programming problem to synchronize the activity of *N* uncoupled cells,

$$\begin{array}{ccc} {\dot{V}}_{k} & = & \tau_{V}^{-1}f\left(V_{k},n_{k}\right)+u\left(t\right),\\ {\dot{n}}_{k} & = & \tau_{n}^{-1}g\left(V_{k},n_{k}\right), \end{array}$$ (5)

for *k* = 1, 2, …, *N*. Here, Eq (5) represents an ODE approximation of multiple uncoupled cells from Eq (1) with *u*(*t*) being a control input applied identically to each cell and *V*_*k*_ and *n*_*k*_ being the transmembrane and gating variables, respectively, of cell *k*. To make this problem more amenable to numerical computation, we first notice that during spiral activity, cells quickly converge to the transient attractor in the absence of any external perturbation. In the absence of external stimulation, this allows us to reduce the dimensionality for each cell to one, with each cell obeying

$$\begin{array}{c} {\dot{\psi }}_{k}=-1, \end{array}$$ (6)

for *k* = 1, 2, …, *N*, where *ψ* represents the cell’s position along the transient attractor, with *ψ* = 0 chosen as a convenient reference point. Eq (6) allows us to understand complicated and potentially high-dimensional dynamics for each cell in terms of a single variable. This strategy has been used extensively in control applications for systems with periodic orbits [37–40], and more recently for excitable systems [33]. We discretize these equations by defining Δ*t* to be the time step and choose *M* points, equally spaced in time by Δ*t* along the transient attractor to adequately cover the initial distribution from the right panel of Fig 2, yielding a control space *ψ*^*d*^ = {0, Δ*t*, 2Δ*t*, …, (*M* − 1)Δ*t*}. Note that *ψ* gives a measurement of how long it will take for the cell to reach position 0 in the absence of any perturbation from diffusive coupling or external stimulus. The possible control values are represented as $U^{d}=\left\{0,1,\cdots ,W\right\}$. We define a discrete state space $X^{d}$ such that it contains a state variable for every possible cell (*ψ*_1_, *ψ*_2_, …, *ψ*_*N*_). It is typical for the size of a dynamic programming problem to grow exponentially with the number of states, and this application is no exception. For instance, in this problem, if we allow *N* cells to occupy any of the *M* locations along the transient attractor, $X^{d}$ would contain *M*^*N*^ unique states, each of which would need to be accounted for on each dynamic programming step. Fortunately because the cells are identical and uncoupled, we can eliminate redundant states by assuming

$$\begin{array}{c} \psi_{1}\geq \psi_{2}\geq \cdots \geq \psi_{N}. \end{array}$$ (7)

The number of possible combinations that need to be considered is equivalent to finding the number of *M*-tuples of non-negtive integers which sum to *N*, which is equivalent to finding the number of weak compositions of *N* with *M* terms. From [41], this reduces the number of states, *S* in the problem to

$$\begin{array}{c} S=\frac{(N+M-1)!}{(M-1)!\;(N)!}. \end{array}$$ (8)

**Fig 3:**
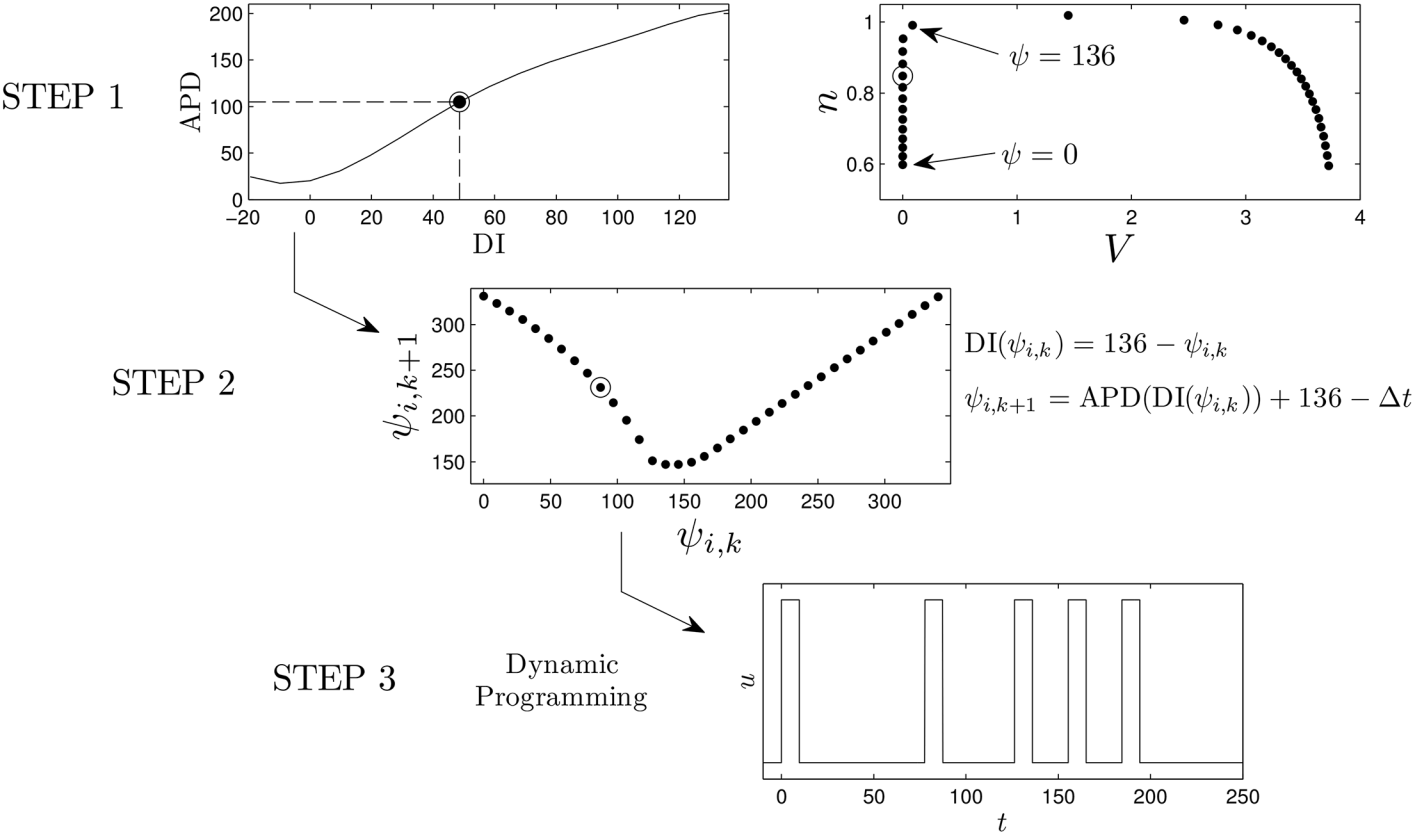
Calculation of optimal pulsing patterns can be obtained in three steps. First, the transient attractor is discretized into points that are equally spaced in time. In the top panel, the cell repolarizes at *ψ* = 136 which corresponds to a DI of zero (i.e. the state at which cell has just repolarized). A mapping based on giving an excitatory stimulus, *ψ*_*i*,*k*_ → *ψ*_*i*,*k*+1_, can be calculated using the equations in step 2. From the numerically determined map using the Karma model, we find that if a cell has been recently reexcited, another stimulus does not affect the time at which the cell repolarizes (i.e. *ψ*_*i*,*k*+1_ = *ψ*_*i*,*k*_ − Δ*t*). In steps 1 and 2, we highlight the information required to calculate the excitation map with circled datapoints. Finally, using the excitation map, the dynamic programming procedure outlined here can be be used to calculate an optimal pulsing pattern. Note that multiple maps for different stimuli can be calculated and included in the optimization procedure.

Using Eq (5), we can formulate the difference equation:

$$\begin{array}{c} x_{k+1}=F\left(x_{k},u_{k}\right)\;\forall k\in \left\{1,2,\cdots ,K\right\}, \end{array}$$ (9)

where $x_{k}\in X^{d}$ is the state of the *N* dimensional, discretized system at time *k*. $u_{k}\in U^{d}$ is the control input at time *k*, *F*(⋅, ⋅) represents the map between states for a given control, and *K* is the end time. We define *u*_*k*_ = 0 to represent the possibility of not giving any external control. From Eq (6), (*ψ*_1_ − Δ*t*, *ψ*_2_ − Δ*t*, …, *ψ*_*N*_ − Δ*t*) = *F*((*ψ*_1_, *ψ*_2_, …, *ψ*_*N*_), 0). If we let *u*_*k*_ = 1 represent a defibrillating pulse, then in order to determine *F*(*x*_*k*_, 1), we simulate a single cell from Eq (5) by applying an excitatory stimulus *u*(*t*) = 2 for 10 ms, and measure the resulting action potential duration. This protocol yields a cellular excitation map, *ψ*_*i*,*k*_ → *ψ*_*i*,*k*+1_, with *i* representing the cell index and *k* representing the discretized time point, shown in step 2 of Fig 3 with *M* = 36 with states indexed clockwise. Note that different choices of *u*(*t*) will produce a similar excitation map provided they are strong enough to produce an action potential. Here, we have chosen *ψ* = 0 to correspond to a cell which has been repolarized for 120 ms. We define repolarization to occur when the variable *V* comes within 95 percent of its resting value (i.e. *V* = *V*_max_ − .95(*V*_max_ − *V*_rest_) where *V*_max_ and *V*_rest_ represent the maximum and resting potentials of the cell, respectively) and choose Δ*t* = 9.7 ms to achieve an adequate discretization. The excitation map can be interpreted as follows: for a cell at *ψ* = 87, an excitatory stimulus will bring the cell to position *ψ* = 231 after time Δ*t* has elapsed. *F*(*x*_*k*_, 1) is calculated by applying the map to each cell, and *x*_*k*_ is reordered to satisfy Eq (7) if necessary.

We seek to find a sequence of *u*_*k*_ which will progressively synchronize the activity of *N* cells along the discretized transient attractor, thereby eliminating spiral wave activity within the myocardium, using the fewest possible number of pulses. We define the time additive cost function:

$$\begin{array}{c} J=\sum_{k=1}^{K}E_{k}+\beta_{p}R\left(x_{K+1}\right), \end{array}$$ (10)

where *β*_*p*_ > 0 is a penalizing scalar and *E*_*k*_ is the energy cost associated with the input *u*_*k*_. Furthermore, *R*(⋅) is chosen to be the standard deviation,

$$\begin{array}{c} R\left(x_{K+1}\right)=\sqrt{\frac{1}{N}\sum_{i=1}^{N}{\left(\psi_{i,K+1}-{\bar{\psi }}_{K+1}\right)}^{2}}, \end{array}$$ (11)

where *ψ*_*i*,*K*+1_ are the states associated with state *x*_*K*+1_, and ${\bar{\psi }}_{K+1}$ is the mean of those states; this is a measure of how synchronized the system is, with lower values corresponding to greater synchrony. Our goal is to find a sequence $u_{k}\in U^{d}$ for *k* = {1, 2, …, *K*} such that Eq (10) is minimized subject to Eq (9).

Following [42], we cast the problem in the dynamic programming format by first defining the cost-to-go function (sometimes referred to as the value function) from a state *x* at time *K* − *q*, denoted $J_{K-q}^{*}\left(x_{K-q}\right)$:

$$\begin{array}{c} J_{K-q}^{*}\left(x_{K-q}\right)=\underset{u_{k}\in U^{d},\forall k\geq K-q}{\inf}\sum_{k=K-q}^{K}E_{k}+\beta_{p}R\left(x_{K+1}\right). \end{array}$$ (12)

According to the principle of optimality [43],

$$\begin{array}{c} J_{K-q}^{*}\left(x_{K-q}\right)=\min_{u_{K-q}}\left\{E_{K-q}+J_{K-(q-1)}^{*}\left(F\left(x_{K-q},u_{K-q}\right)\right)\right\}. \end{array}$$ (13)

Eq (13) is a recurrence relation that allows us to iteratively calculate the cost-to-go function, starting with the end point cost, *R*(*x*_*K*+1_), and working backwards. Once the cost-to-go function is obtained, the optimal control and trajectory are

$$\begin{array}{c} u_{k}^{*}=\text{arg}\min_{u_{k}\in U^{d}}\left(E_{k}+J_{k+1}^{*}\left(F\left(x_{k}^{*},u_{k}\right)\right)\right), \end{array}$$ (14)

$$\begin{array}{c} x_{k+1}^{*}=F\left(x_{k}^{*},u_{k}^{*}\right), \end{array}$$ (15)

for *k* ∈ {1, 2, …, *K*} starting from an initial state *x*_1_. The steps involved in the formulation and calculation of an optimal series of pulses are summarized in Fig 3.

**Fig 4:**
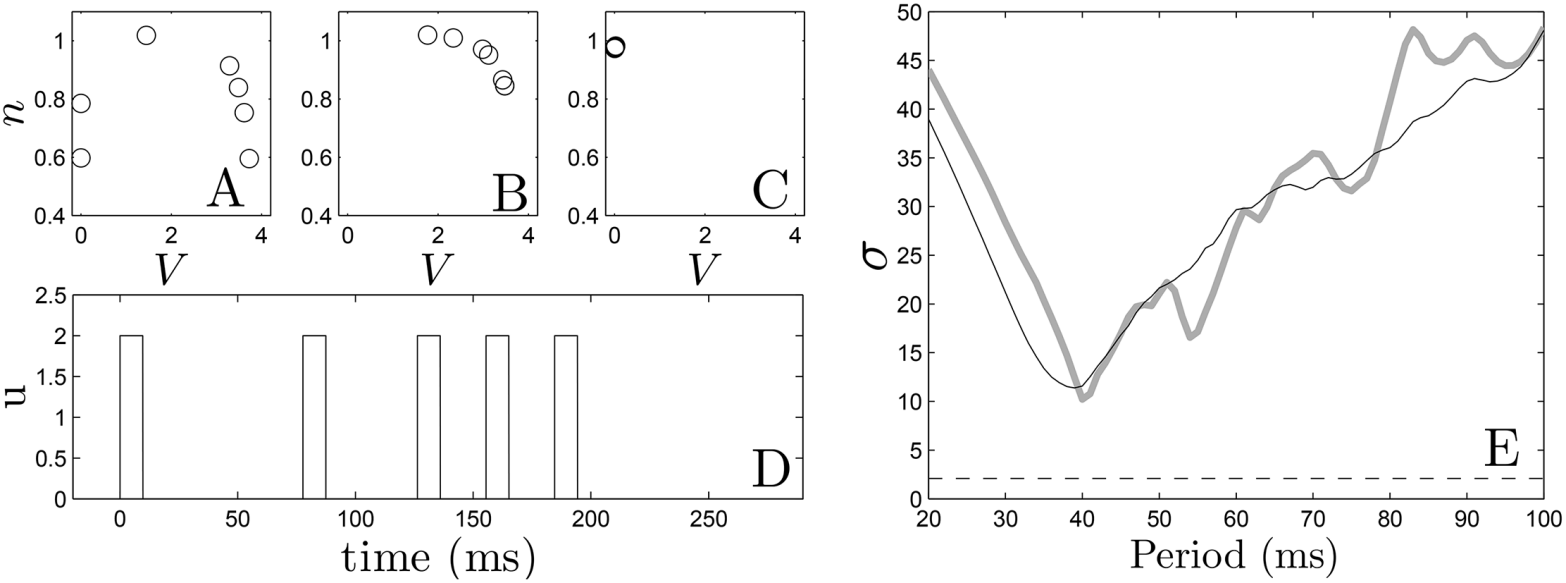
Panels (A)-(C) show the optimal states of the 7 cell system at times 0, 100, and 220 ms, respectively. Panel (D) shows the optimal pulse train obtained from dynamic programming. In panel (E), we apply five pulses at constant period to the same system of 7 cells (grey line) and a system with one cell at each of the 36 states (black line) along the transient attractor as initial conditions. We see qualitative agreement between both plots. The dashed line represents the standard deviation obtained from optimal pulse train on the 7 cell system.

**Fig 5:**
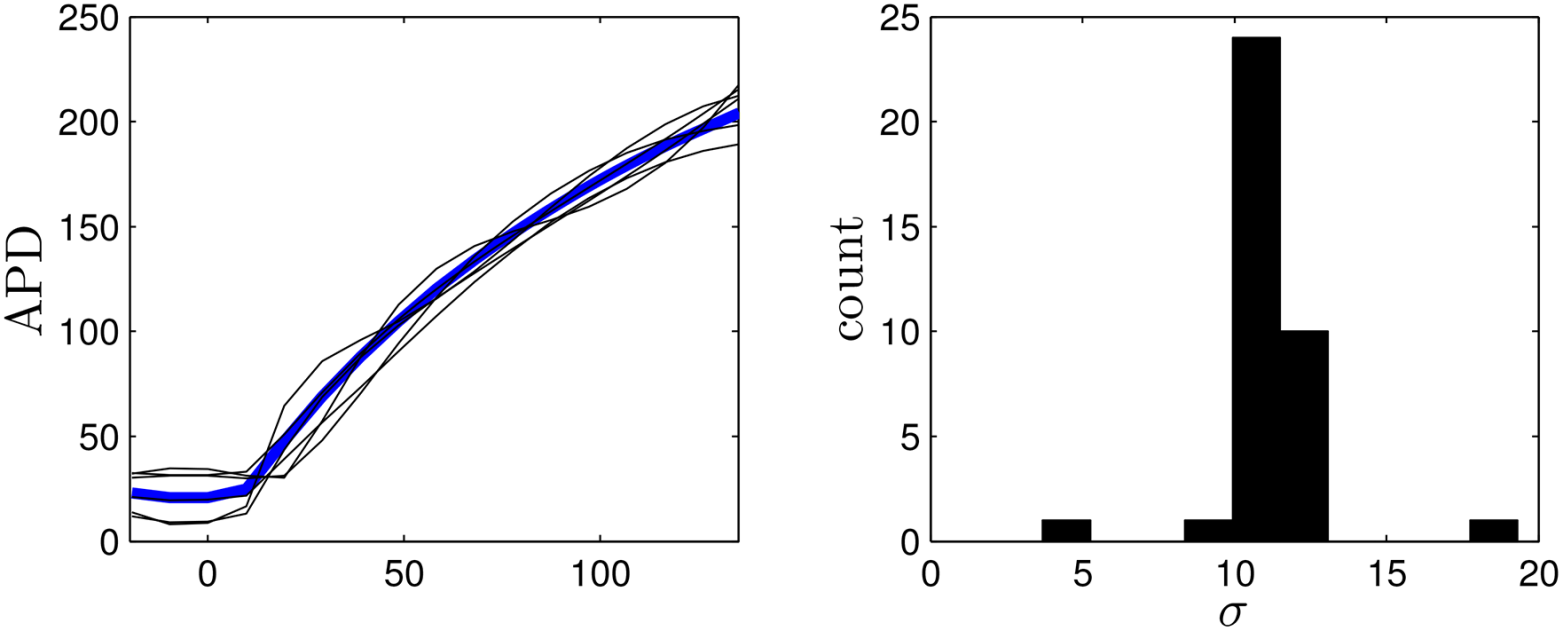
The left panel shows the true APD restitution curve for the Karma model in blue. The optimization process is performed on other randomly generated curves to gauge the robustness of the optimization algorithm. Five representative curves are shown as black lines. The right panel shows the synchronization from resulting optimal stimuli applied to 36 uncoupled cells reported as the standard deviation of their repolarization times.

In the dynamic programming algorithm, a larger (resp. smaller) choice of *E*_*k*_ relative to *β*_*p*_ will yield an answer with fewer (resp. more) pulses. For the Karma model, we choose *β*_*p*_ = 1.5, and choose 2.4 to be the cost of giving an excitatory pulse. We note that in this application, we only have two options: giving an excitatory pulse or not giving a pulse. We choose an end time of *K* = 30 corresponding to *t* = *K*Δ*t* = 291 ms. Ideally we would like to include as many cells as possible in the dynamic programming problem, but computational memory considerations limit the number of cells to *N* = 7 while maintaining an adequate discretization. Panel (D) of Fig 4 shows the resulting optimal sequence. Note that while this sequence is optimal, it is not necessarily guaranteed to be unique (i.e. multiple sequences which are all globally optimal could exist). We also note that we do not choose the optimal pulse train to contain five pulses *a priori*, but rather, this results from the parameters chosen in the optimization process. We see that this optimal pulse train begins with pulses that are relatively spread out, and ends with more frequent pulses. Panels (A)-(C) show the state of the system at times 0, 100, and 220 ms, respectively. Progressive synchronization of the uncoupled cells is clearly demonstrated, and gives a standard deviation *σ* = 2.1 of the times at which cells become repolarized. For comparison, we also simulate trains of 5 pulses with a constant period and the same pulse height as the optimal sequence, with results shown in Panel (E). We find that the optimal sequence synchronizes the cells better than any periodic pulse train as measured by the standard deviation of the times that the cells become quiescent. Because the optimal pulsing sequence takes the APD restitution curve as its primary input, we would expect the optimal pulsing pattern to be sensitive to errors in its measurement. To give a sense of the effect of these errors on the resulting pulsing pattern, we apply the optimization method using APD restitution curves which deviate from the true curve, with examples given in Fig 5. Each curve is obtained by taking the true APD curve, adding a random number to each data point, and fitting the resulting points to a fourth order polynomial. The resulting pulsing patterns are applied to a population of 36 uncoupled cells with different initial conditions equally spaced in time along the transient attractor. For 37 trials which produce optimal patterns with five pulses, the average standard deviation in repolarization times is 11.1. Compared with the results from Fig 4, this performance is on par with the best result that can be obtained using fixed interval pulses.

We apply the optimal pulse train to a 2-dimensional bidomain Model (1) with the ionic currents defined for each cell according to the Karma Model (5). We take a relatively large square grid with side length 50.5 cm so that it can support a large number of spiral waves. The period of each spiral wave is approximately 150 to 170 ms, depending on its interaction with the conductivity discontinuities. We compare the optimal strategy to a single pulse strategy, in addition to a fixed-time pulsed strategy with a period of 40, 60, and 200 ms and a single pulse strategy. The duration of all pulses was 10 ms. The 40 ms period was chosen as the best synchronizing rate from panel E of Fig 4, with the 60 and 200 ms pulsing period chosen for comparison. From panel (E) of Fig 4, we would expect the series of pulses with 40 ms period to synchronize the tissue more effectively than the series of pules with 60 ms period, resulting in a higher rate of successful defibrillation. For an excitatory stimulus in the bidomain equations, we apply a voltage gradient across the tissue. We take *C*_*m*_ = 2*μF*/cm^2^, *β* = 1000cm^−1^ and *g*_*α*_ to be 0.8, 2, 0.2, 2, 0.8 and 0.2 mS/cm for *α* = *ex*, *ey*, *ix*, *iy*, *ox*, *oy*, respectively These anisotropy ratios are consistent with those reported in [44]. Simulations are performed on a 320 × 320 grid.

**Fig 6:**
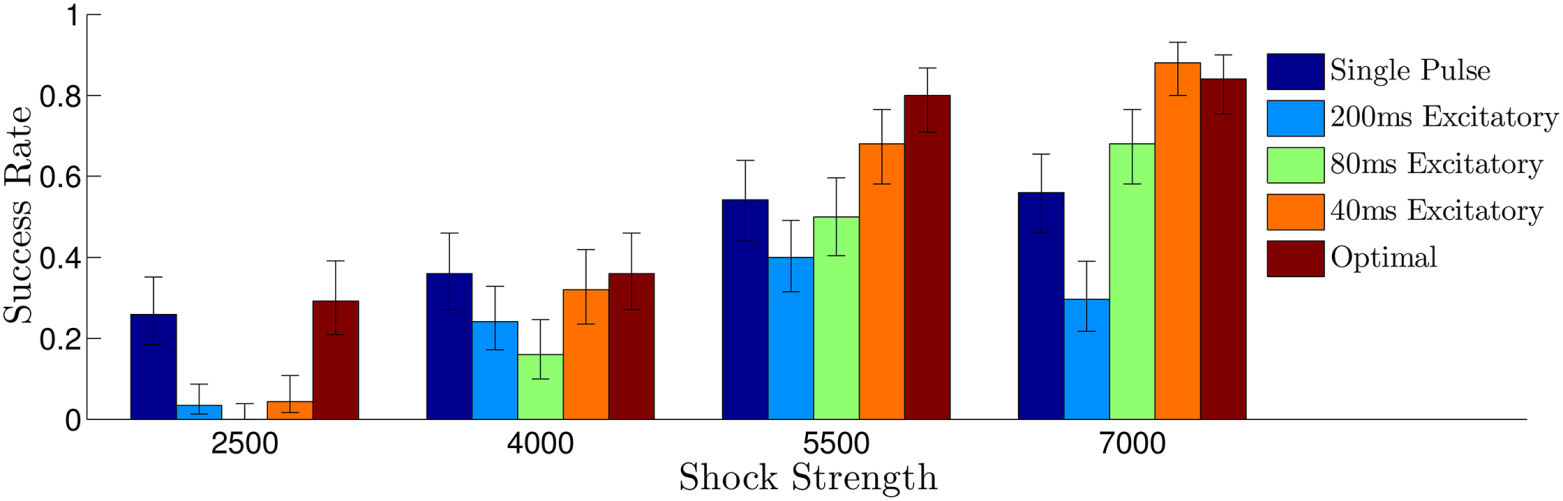
Comparison of the success rate for various defibrillation strategies. Error bars represent a confidence interval corresponding to one standard deviation. Overall, the synchronization predicted from panel E of Fig 4 is correlated to the success rate for each shock strategy. For all multiple pulse trials, the shock strength is reported as the difference between the maximum and minimum extracellular voltages. For the single defibrillating pulse, the induced voltage gradient is $\sqrt{5}\times \left(\text{Shock}\;\text{Strength}\right)$ to keep energy consumption equivalent to that of the other trials, assuming energy consumption is proportional to ∫ (Shock Strength)^2^ *dt*.

For simulations of the Karma model, we randomly remove 650 sets of gap junctions with a maximum, minimum, and average length in the principal fiber direction of 2.8, 0.5, 1.7 percent of the domain, respectively (see the top panel of Fig 1). Bidomain simulations of Eq (1) were performed using a fully explicit forward Euler scheme (described by Eqs (14) and (15) of [45]). Linear systems from the resulting discretization were solved with a generalized minimal residual algorithm using the CUSP package [46]. For each trial, the system is simulated long enough so that initial transients due to the initiation of the spiral waves die out, and we categorize the defibrillation as successful if all spiral waves are eliminated by the excitatory pulses. Each trial uses different initial conditions with a different random set of gap junctions removed. Results are plotted in Fig 6, with error bars corresponding to one standard deviation calculated from a Wilson score interval with at least *n* = 23 trials for each data point. For the multiple pulse strategies, shock strengths are reported as the maximum difference in extracellular voltage over the entire 2D domain during an excitatory pulse. For the single defibrillating pulse, the shock strength is chosen so that the energy consumption is identical to the multiple pulse strategies, assuming that energy consumption of each shock is proportional to ∫ (Shock Strength)^2^ *dt*. We find that the optimal strategy outperforms the single, 60 ms, and 200 ms period strategies at all shock strengths, and is better than the 40 ms period strategy at low energies. We note that while the success rate of 40 ms period pulsing strategy was similar to the optimal strategy, we were only able to determine that a 40 ms pulsing period worked well for synchronizing the cells through trial and error (see Fig 4). This trial and error approach is simple *in silico*, but may be much more difficult in real tissue. Conversely, the calculation of an optimal stimulus only requires a few measurements to determine the excitation map *ψ*_*i*,*k*_ → *ψ*_*i*,*k*+1_.

**Fig 7:**
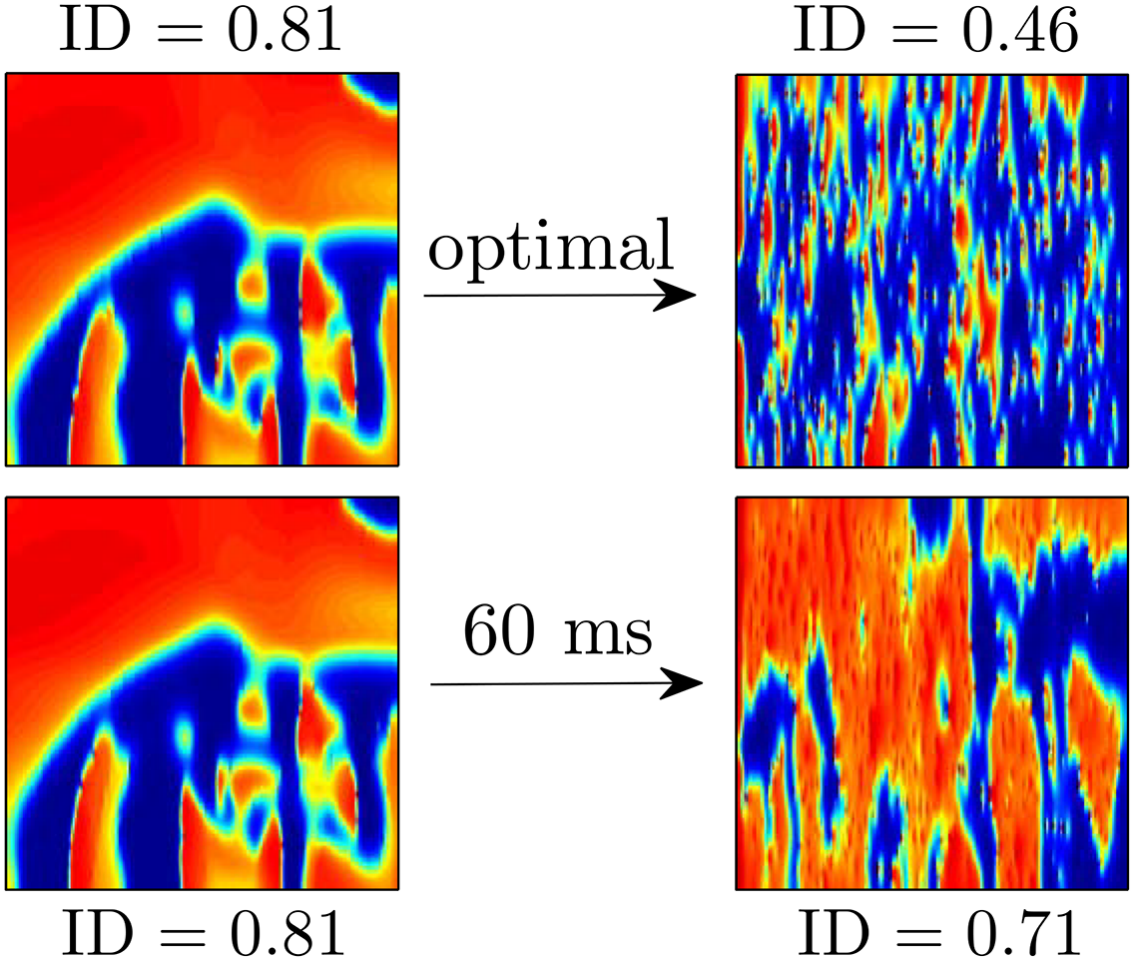
For the Karma model, the ID metric given in Eq (16) gives a sense of the spatial synchronization in the tissue. In the top panels, using the optimal stimulus yields an ID of 0.46 40 ms after the final pulse is applied. The distribution of excited and quiescent cells is similar throughout the tissue, making it less likely that the remaining wave fronts will find a reentrant pathway. In the bottom panel, using the 60 ms pulsed stimulus yields an ID of 0.62 40 ms after the final pulse is applied. The large connected regions of excited and quiescent tissue make it more likely that the remaining wave fronts will produce new reentrant waves.

Our original hypothesis was that increased synchronization would make spiral wave reentry less likely. For this particular model however, regardless of the pulsing pattern, excitatory shocks produce a similar pattern of depolarization and hyperpolarization throughout the medium due to the virtual electrodes. For this reason, a metric that only examines synchronization has similar features regardless of the pulsing pattern used. Instead, for this model we choose a measurement of how well the excited and quiescent tissue is mixed throughout the tissue. If the excited and quiescent tissue is well mixed after the final pulse, then the series of pulses was successful at synchronizing a large majority of tissue, and the pattern in the 2D medium is primarily due to locations at which the influence of the virtual electrodes was particularly strong. Conversely, if regions which were similar in location along the transient attractor before the pulses started are still similar after the final pulse, then the excited and quiescent tissue will not be well mixed, making spiral reentry more likely (see Fig 7 for illustration). Therefore, to give a sense of the spatial heterogeneity in the tissue we track the index of dissimilarity [47] (ID) throughout the tissue:

$$\begin{array}{c} \text{ID}\left(t\right)=\frac{1}{2}\sum_{j=1}^{N}|\frac{e_{j}\left(t\right)}{E(t)}-\frac{q_{j}\left(t\right)}{Q(t)}|. \end{array}$$ (16)

**Fig 8:**
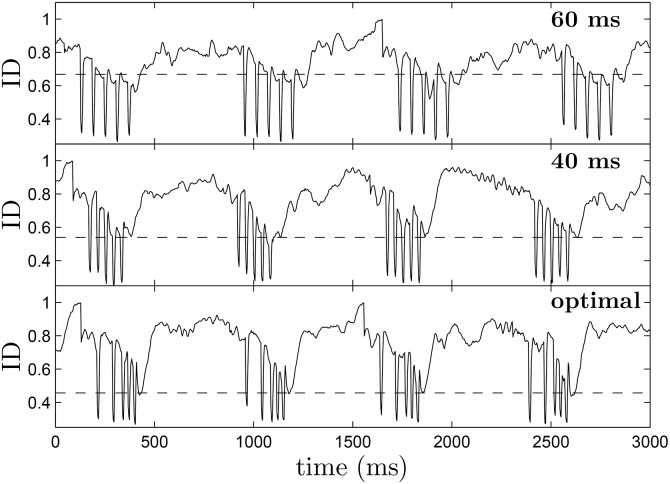
The top, middle, and bottom panels show representative plots of the ID as a function of time over multiple trials using the 60 ms pulsed, 40 ms pulsed, and optimal pulsing strategies, respectively. During each pulse, the ID drops suddenly, because of the pattern of hyperpolarization and depolarization that the virtual electrodes create. If the ID soon after the final pulse is small, it indicates that excited and quiescent tissue are thoroughly mixed, making it less likely for spirals to reenter. Over multiple trials, the average value of ID 30 ms after the final pulse is 0.456, 0.539, and 0.668 for the optimal, 40 ms pulsed, and 60 ms pulsed strategies, respectively, as indicated by dotted lines in each figure. These results are consistent with the synchronization that would be expected from panel (E) of Fig 4.

To calculate Eq (16), the 2-dimensional tissue, which is on a 320 × 320 grid, is divided into *N* = 16^2^, 20 × 20 bins, where *e*_*j*_(*t*) and *q*_*j*_(*t*) represent the number of excited and quiescent cells in region *j*, respectively, and *E*(*t*) and *Q*(*t*) represent the total number of excited cells and quiescent cells at time *t*. Cells are categorized as excited if *V*_*m*_ > 1, and deemed quiescent otherwise. The ID can be understood intuitively as the proportion of excited cells which would need to be redistributed so that the distribution in each bin matches the global distribution; a small value of ID means that the quiescent and excited cells are well mixed throughout the tissue. Fig 8 shows the ID plotted over multiple trials using each strategy when the shock strength is 50 percent of the maximum. Horizontal dotted lines represented the average value of ID 30 ms after the final pulse has been applied. We find that before pulses are applied ID is close to 0.8 when spiral waves are present in the medium. While ID drops suddenly during each pulse because of the pattern of hyperpolarization and depolarization that occurs at each discontinuity, it is important to consider the value that the ID rebounds to as a measure of the dissimilarity throughout the tissue. On average, the ID 30 ms after the final pulse has been applied is significantly lower for the optimal stimulus than it is for the 40 or 60 ms pulsed strategies. Intuitively, a more heterogeneous distribution of quiescent and excited tissue, makes it is less likely that spirals will be able to find a pathway to reenter the tissue after the pulses are finished.

**Fig 9:**
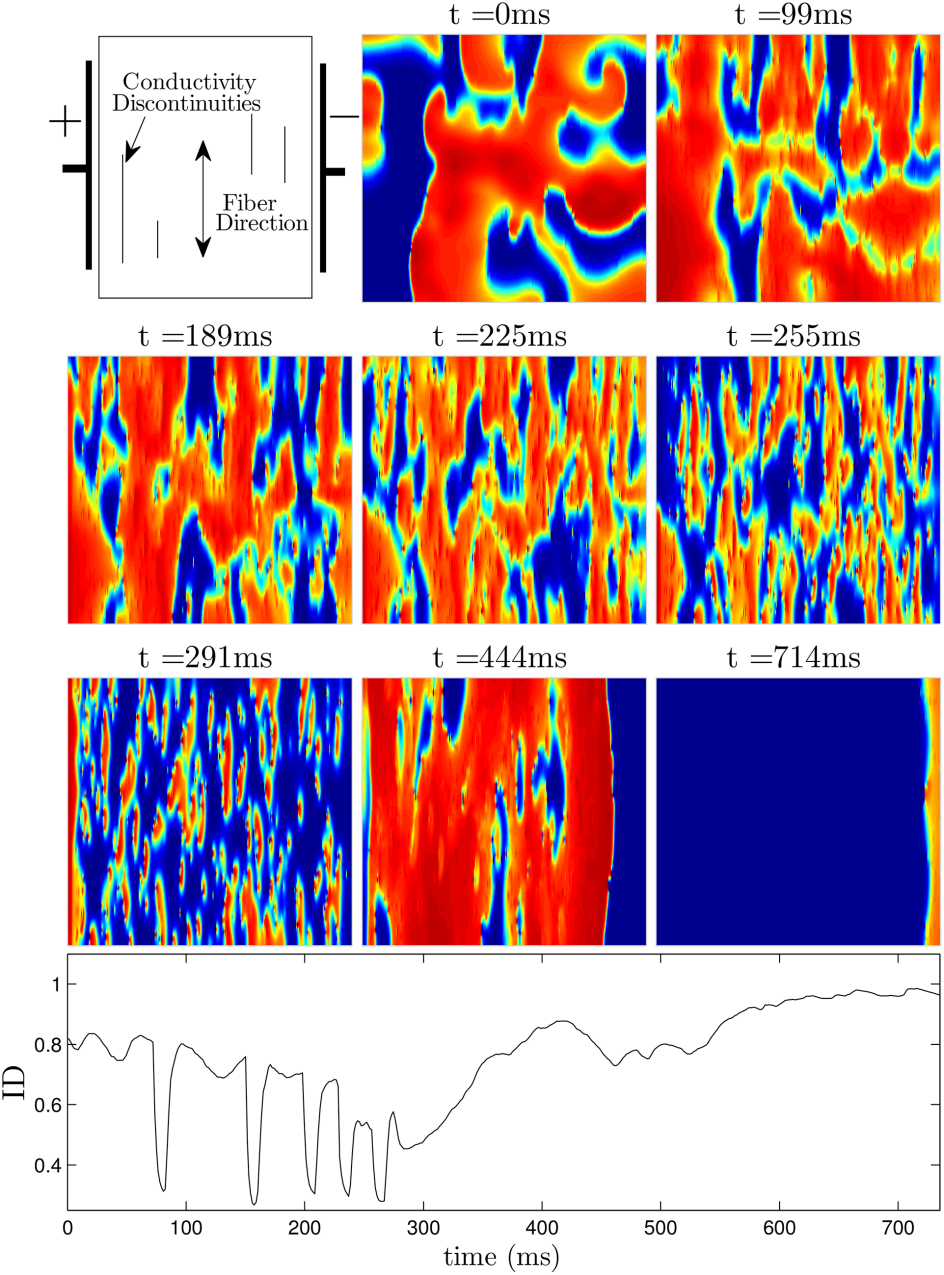
Successful defibrillation using a 5-pulse optimal strategy. The panel at *t* = 0ms shows the system before a defibrillating stimulus is applied. The next five panels show the system approximately 30ms after successive pulses are applied. Notice that soon after the last pulse is applied at *t* = 291ms, the ID is close to 0.45, indicating that the excited and quiescent cells are spread evenly throughout the medium. In the final two panels, any remaining spiral waves are extinguished. The bottom panel shows ID as a function of time. See also S2 File.

**Fig 10:**
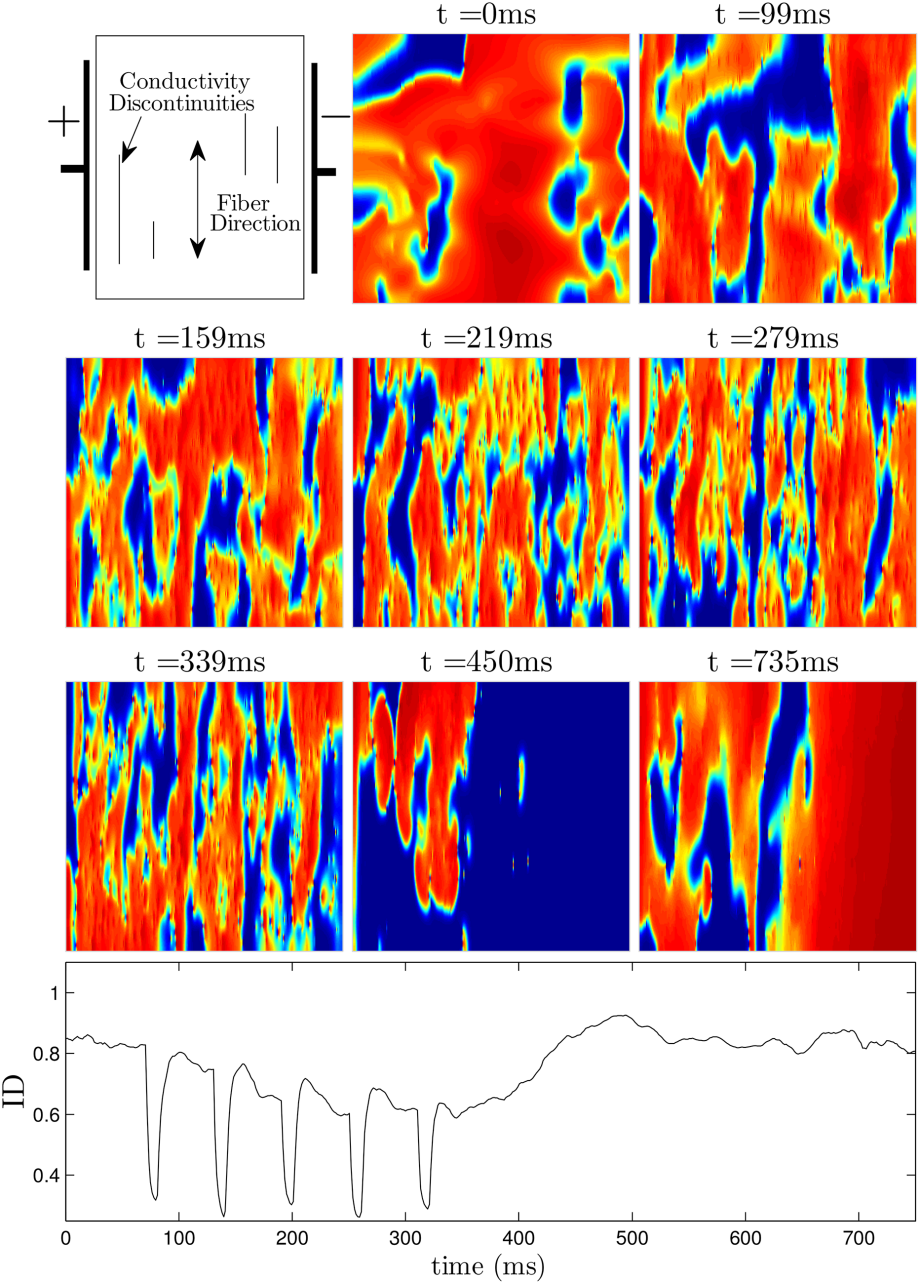
Unsuccessful defibrillation using five pulses, applied 60 ms apart. The panel at *t* = 0ms shows the system before a defibrillating stimulus is applied. The next five panels show the system approximately 30ms after successive pulses are applied. Notice that soon after the last pulse is applied at *t* = 339ms, the ID is around 0.65, indicating that there will likely be pathways for spiral waves to reenter, as shown in the final two panels. See also S3 File.

Figs 9 (see also S2 File) and 10 (see also S3 File) show defibrillation attempts for the optimal and 60 ms period pulsed strategies at 50 percent of the maximum shock strength. The panel at *t* = 0 in each figure shows the system before any defibrillating pulses are applied. The next five panels show the system approximately 30 ms after each pulse is applied. Note that after the final pulse at *t* = 291ms in Fig 9 (corresponding to the optimal pulse train), any remaining excited cells are spread evenly throughout the medium, while after the final pulse at *t* = 339ms in Fig 10, large regions in the domain remain depolarized, while other regions are quiescent. This spatial heterogeneity is conducive to spiral reentry, as can be seen in the final two snapshots of Fig 10. The final two snapshots in Fig 9 show that activation fronts quickly depolarize most of the cells in the medium, terminating any remaining spiral waves.

Finally, we briefly discuss the feasibility of using biphasic defibrillation [48, 49] strategies, which use secondary shocks to reverse the flow of current caused by the first shock. Biphasic shocks have been shown to require less energy to defibrillate than monophasic shocks. The preceding optimization calculations were performed with the assumption that each defibrillating pulse creates virtual electrodes that depolarize tissue in an all-or-nothing fashion. We will assume that a secondary pulse with opposite sign will not create or remove any virtual electrodes, but will simply remove residual charge that may be left over from the primary pulse [50, 51]. With these assumptions, the optimization calculations yield the same optimal pulsing sequence. In pilot simulations, biphasic shocks consisted of a pulse with a shock strength of 4000 (resp. 5500) followed immediately by a pulse with shock strength -2000 (resp. -2750). Here the secondary negative perturbation lasts twice as long as the primary positive perturbation. Using the optimal pulsing pattern yielded a success rate of 86 percent over 21 trials (resp. 100 percent over 29 trials). Using five identical biphasic perturbations spaced 40ms apart yielded a success rate of 50 percent over 20 trials (resp. 76 percent over 29 trials). Compared to the results presented in Fig 6, these preliminary results suggest that a biphasic waveform might further increase the efficacy of an optimal pulsing pattern. A comprehensive study using biphasic waveforms is beyond the scope of this work.

## Control Strategy Applied to the ten Tusscher-Panfilov Model of Cardiac Activity

**Fig 11:**
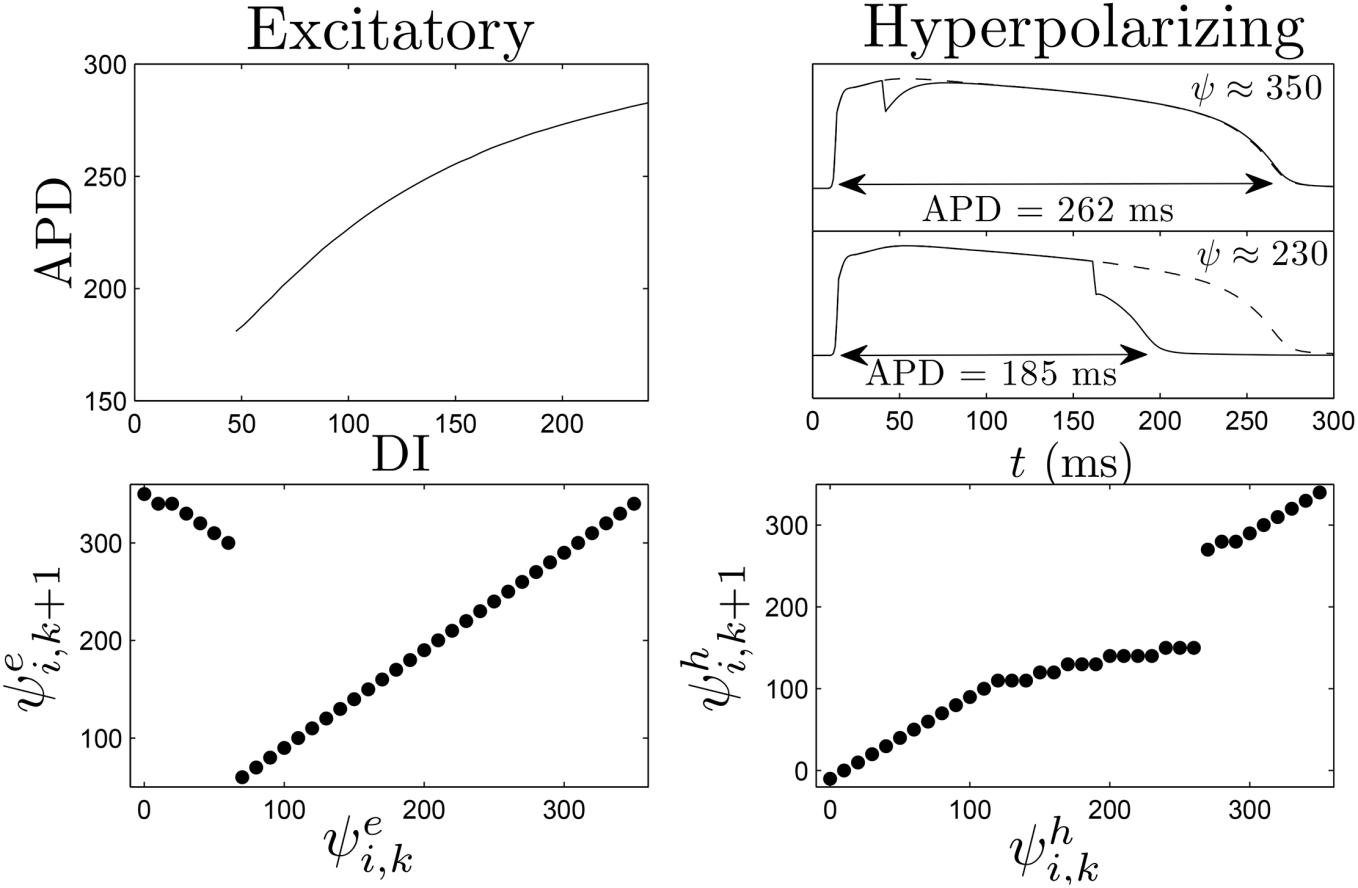
The top-left panel shows the APD restitution curve DIs, and the bottom-left panel shows the excitation map, which is inferred from the APD restitution curve. The top-right shows that a strictly hyperpolarizing stimulus applied soon after a cell is excited (*ψ* ≈ 350) has little effect on the time at which the cell repolarizes, while the same stimulus applied later (*ψ* ≈ 230) will hasten repolarization. The dashed line shows the transmembrane voltage if the strictly hyperpolarizing stimulus had not been applied. The bottom-right panel shows the hyperpolarization map, which is calculated for each discretized phase.

We now illustrate our control strategy for the ten Tusscher-Panfilov model [52]. This model is based on experimental data for human ventricular cells and includes important intracellular ionic currents. We use the parameter settings from [52] which yield an APD restitution curve with a slope of 1.4 when measured with a single cell, and use a Rush and Larsen [53] integration scheme to integrate the Hodgkin-Huxley type gating variables. In tissue, the spiral period is approximately 230 to 250 ms. We measure the APD restitution curve shown in the top left panel of Fig 11 using an *S*1 − *S*2 pacing protocol with *S*1 = 230 ms: the tissue is paced at 230 ms until the dynamics achieve a steady state, the next pulse is presented at a given DI, and the APD is measured to yield a single data point. In order to pace the tissue, we use direct current stimulation at a point source in the center of the tissue with *I*_stim_/*C*_*m*_ = 150 *μ*A/*μ*F. The excitation map can be calculated from the resulting APD restitution curve, measured with respect to a location near the edge of the tissue. In this example we let *ψ* ∈ [0, 350], where *ψ* = 0 represents the time 120 ms after the cell repolarized. Repolarization is defined to occur when the transmembrane voltage comes within 95 percent of its resting value (i.e. *V*_max_ − .95(*V*_max_ − *V*_rest_)), and take Δ*t* = 10. If we excite a cell at *ψ* = 0, the resulting APD will be approximately 240 ms, and 10 ms after the excitation, *ψ* ≈ 350. This process can be repeated for each 10 ms bin, to determine the excitation map, shown in the bottom-left panel of Fig 11. Note that if *ψ* > 70 (corresponding to DI<50), the cell is not excitable, and *ψ* simply decreases by 10 ms after an attempted excitation. We note that the range of *ψ* and its discretization can be taken slightly differently in the dynamic programming algorithm to yield qualitatively similar results.

For this model, as might be expected from the excitation map, we find that it is not possible to design a pulse train of excitatory pulses to synchronize the tissue because there is a very small window in which each cell can be excited. One possibility of mitigating this problem would be to extend the maximum allowable DI, but the resulting optimal pulse trains would contain some pulses which are spaced close to the natural spiral period, invalidating the assumptions of our control algorithm. In this example, in order to illustrate the power of the proposed control methodology and to design a stimulus which can significantly synchronize the cells, as a second stimulus option we include the possibility of giving a strictly hyperpolarizing stimulus through current injection uniformly to each cell. We note that a strictly hyperpolarizing stimulus is not plausible in a clinical setting, and include the possibility of a hyperpolarizing stimulus to showcase the modularity of the proposed control algorithm; including different types of external stimuli can be readily handled by the control methodology and can greatly improve synchronization of the tissue. We also emphasize that the control methodology is not limited to only the stimuli we consider in this manuscript, and could be implemented with another stimulus provided that its effect can be characterized by a map of the form *ψ*_*i*,*k*_ → *ψ*_*i*,*k*+1_. With this in mind, we assume that we have the ability to give uniform strictly hyperpolarizing pulses throughout the entirety of the tissue, *I*_stim_/*C*_*m*_ = −70*μ*A/*μ*F, for 1 millisecond. To measure the hyperpolarization map, one can give the strictly hyperpolarizing stimulus at a known phase, and determine the time at which the cell repolarizes. For instance, when stimulus is applied at *ψ* ≈ 230, the cell repolarizes approximately 30 seconds later. Recall that *ψ* = 120 corresponds to the time a cell repolarizes, therefore *ψ* ≈ 120 + 30 − Δ*t* = 140 ms after the stimulus is applied. The hyperpolarization map can then be used in the optimization.

**Fig 12:**
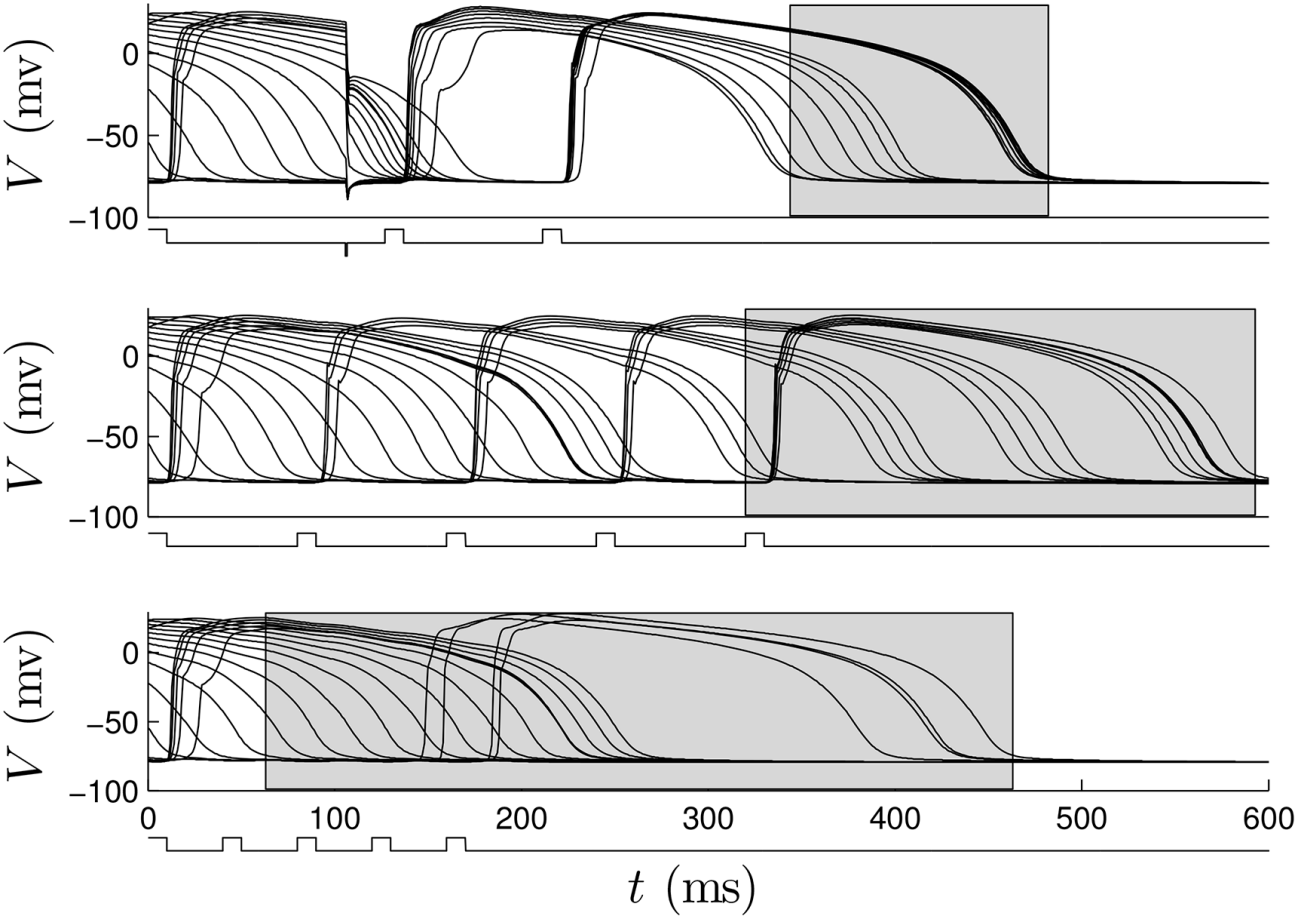
The top, middle, and bottom panels show 18 cells with equal initial spacing in *ψ* with optimal, 80 ms pulsed, and 40 ms pulsed control strategies applied, respectively. The grey boxes represent the distribution of times at which cells repolarize. In the signals below the voltage traces, positive pulses represent excitatory perturbations. The negative pulse in the top panel occurs at approximately 100 ms and represents a strictly hyperpolarizing perturbation.

For the dynamic programming calculations, we choose *β*_*p*_ = 0.75, and choose 2.4 to be the cost of giving either an excitatory or strictly hyperpolarizing pulse. We choose an end time of *K* = 40 corresponding to *t* = 400. The resulting optimal control contains three excitatory pulses and one inhibitory pulse. In S4 File, we apply this control to a group of 18, 2 dimensional patches of tissue, with initial conditions equally spaced in *ψ*. Here, the patches are not coupled to each other. In these simulations, during an excitatory stimulus, depolarizing current is applied to a small region near the middle of the patch to mimic the wave of spreading depolarization created from a virtual electrode. The resulting transmembrane voltage is shown for a single cell from each patch in the top panel of Fig 12. We compare this strategy to a 80 ms period (see S5 File) and 40 ms period excitatory pulsed strategy, shown in the middle and bottom panels of Fig 12. Note that for this model, the synchronization in individual cell simulations did not depend strongly on pulsing periods in the range between 30 ms and 100 ms. We find that the optimal strategy greatly outperforms the other strategies in terms of the distribution of times at which the cells repolarized, as represented by the grey boxes.

**Fig 13:**
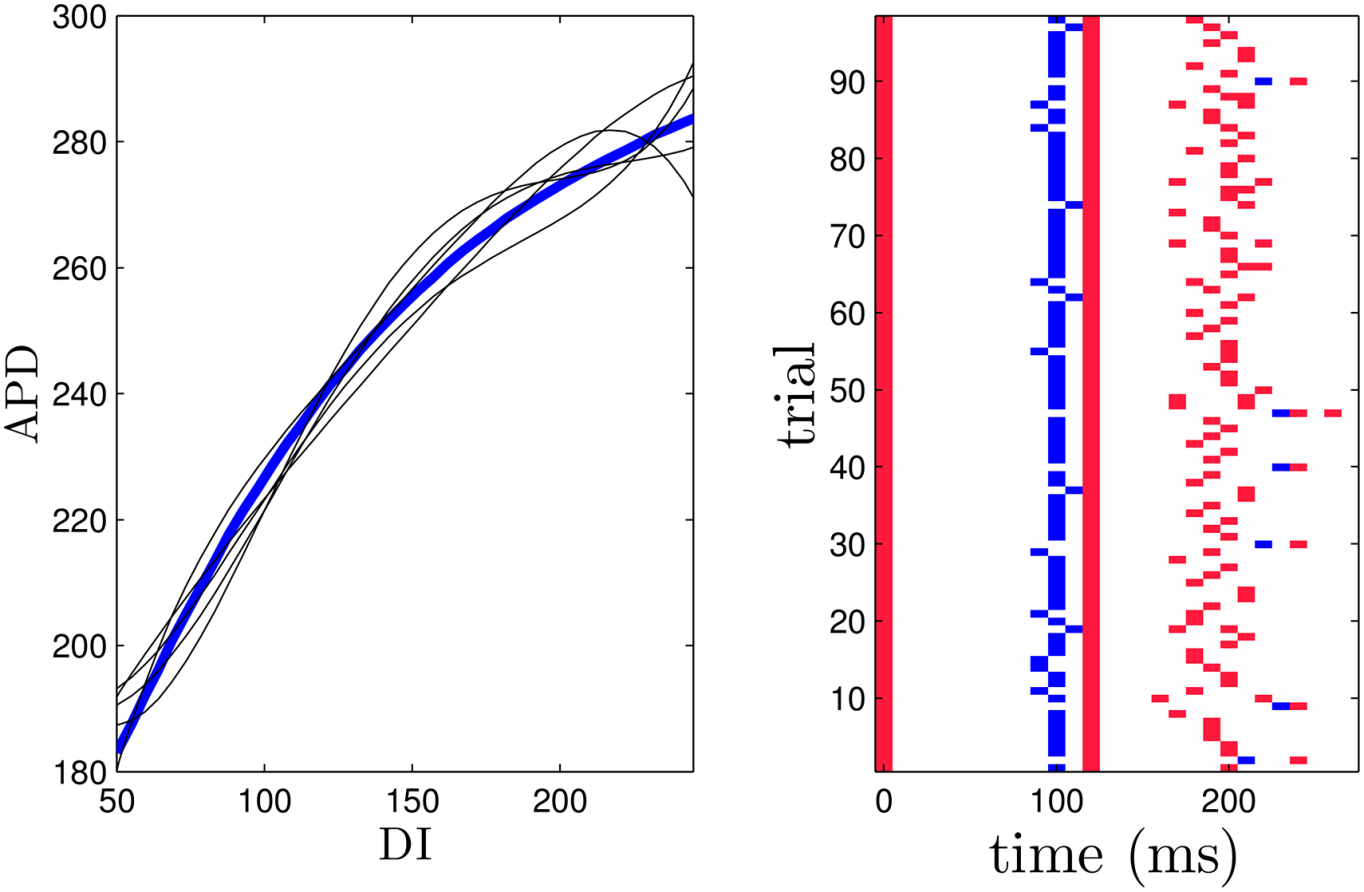
The left panel shows the true APD restitution curve for the ten Tusscher-Panfilov model in blue. The optimization is performed using randomly modified APD restitution curves and hyperpolarization maps as described in the text. Five representative APD curves are shown in black. The right panel gives the resulting optimal pulsing pattern calculated over 98 trials. Red marks indicate excitatory pulses and blue marks represent hyperpolarizing pulses. For comparison, the optimal pulse series using the true data has excitatory pulses at 0, 120, and 200 ms and a hyperpolarizing pulse at 100 ms.

In this optimization, errors in measuring both the effect of the depolarizing and hyperpolarizing pulses will contribute to errors in the resulting optimal pulsing pattern. As in the previous example, we give a sense of the robustness of these calculations by applying the optimization algorithm when the input data is not perfect. Multiple excitation maps were obtained using APD restitution curves which are different from the true restitution curve. Each APD restitution curve curve is obtained starting with the true APD curve, adding a random number to each data point, and fitting the resulting points to a fourth order polynomial. Representative curves used in the optimization procedure are given in the left panel of Fig 13. Errors are also introduced to the hyperpolarization map for all data points corresponding to times when the cell has not yet repolarized, (i.e. for isostable values greater than 120) by starting with true data points and adding a randomly chosen number drawn from a normal distribution with mean zero and standard deviation 15. The right panel of Fig 13 shows the resulting optimal stimuli over 98 trials. The resulting pulsing patterns are qualitatively similar to the true optimal series of pulsing with slight variations in timing depending on the inputs to the optimization. Occasionally the hyperpolarizing pulse is applied later in the resulting pulse sequence, but is always followed soon after by an excitatory pulse.

Finally, we apply the optimal pulsing strategy to the 2-dimensional bidomain Model (1) on a square domain with side length 4.16 cm. We also compare this strategy to an excitatory fixed pulsed strategy with a 40, 80, and 200 ms period and a single pulse strategy. We also compare to a non-optimal strategy implemented with a strictly hyperpolarizing stimulus followed by three excitatory stimuli at 80 ms intervals. In the bidomain equations, for an excitatory stimulus, we apply a constant voltage gradient across the tissue, and when a strictly hyperpolarizing pulse is applied, *I*_stim_ = 70*μ*A/cm^2^ for each cell. We take *g*_*α*_ to be 0.214, 0.375, 0.0375, 0.375, 0.214, and 0.375 S/m for *α* = *ex*, *ey*, *ix*, *iy*, *ox*, *oy*, respectively, [54]. We also take *β* = 1000 cm^−1^. Here, we randomly remove 400 sets of gap junctions with a maximum, minimum, and average length of 2100, 300, and 1200 microns, respectively, oriented along the fiber direction by taking *g*_*ix*_ = 0. All other parameters are the same as those given in [52]. Bidomain simulations of Eq (1) were performed on a 320 × 320 grid using forward Euler with a generalized minimal residual algorithm. For these simulations, the system is simulated long enough so that initial transients due to the initiation of the spiral waves die out and we categorize the defibrillation as successful if all spiral waves are eliminated by the excitatory pulses. Each trial uses different initial conditions with a different random set of gap junctions removed.

**Fig 14:**
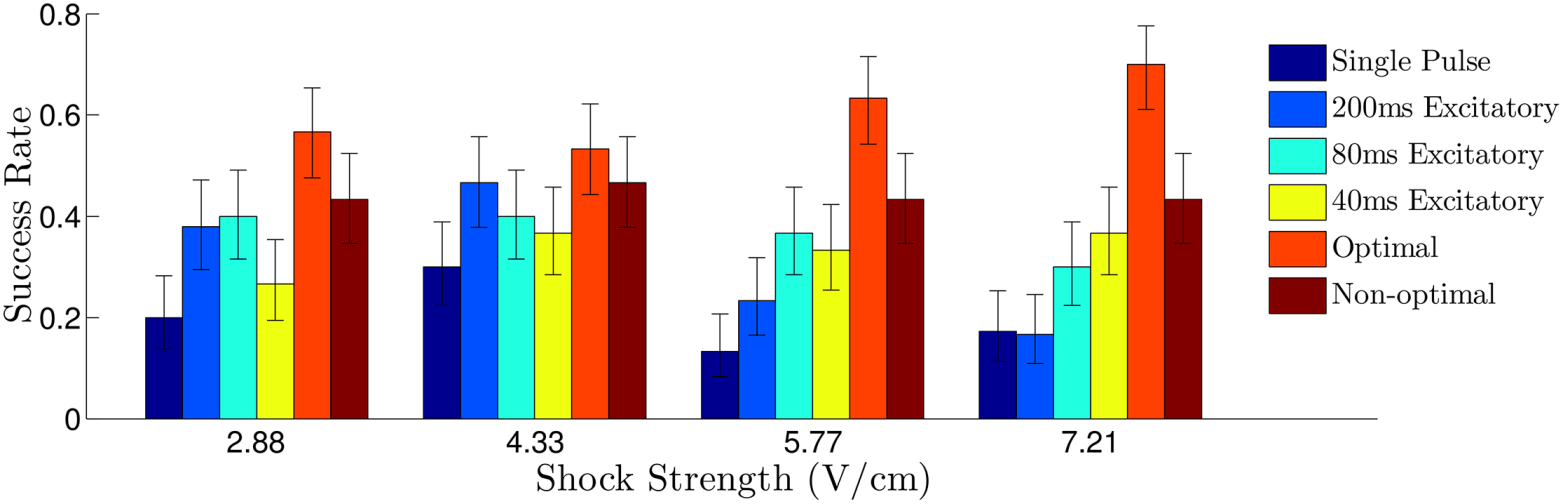
Numerically observed probability of successful defibrillation of the ten Tusscher-Panfilov Cardiac Model. Error bars represent a confidence interval corresponding to one standard deviation. For all multiple pulse trials, the shock strength is reported as the voltage gradient induced during an excitatory pulse. For the single defibrillating pulse, the induced voltage gradient is $\sqrt{5}\times \left(\text{Shock}\;\text{Strength}\right)$ to keep energy consumption equivalent to that of the strictly excitatory trials, assuming energy consumption is proportional to ∫ (Shock Strength)^2^ *dt*.

**Fig 15:**
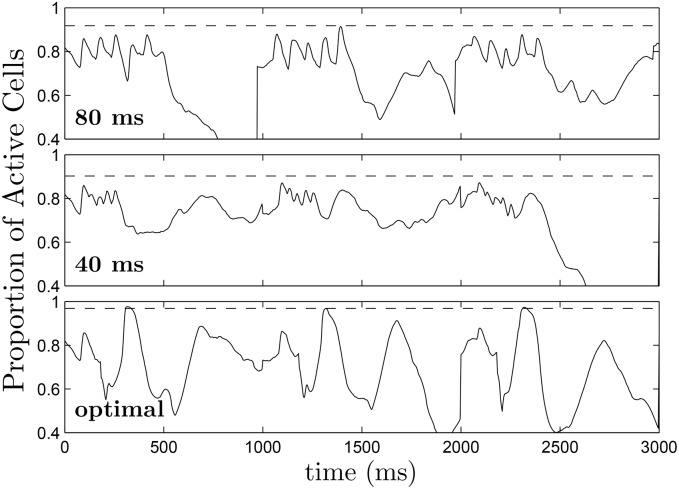
The top, middle, and bottom panels show representative plots of the proportion of active tissue (transmembrane voltage greater than -70 mV) as a function of time over multiple trials using the 80 ms pulsed, 40 ms pulsed, and optimal strategies respectively with an excitatory pulse strength of 2.89 V/cm. Dashed lines at 0.968, 0.903, and 0.918 for the optimal, 40 ms pulsed, and 80 ms pulsed strategies, respectively represent the maximum proportion of active cells, averaged over multiple trials.

Results are plotted in Fig 14, with error bars corresponding to one standard deviation calculated from a Wilson score interval with at least *n* = 29 trials for each datapoint. In this case, we find that the optimal strategy outperforms each pulsed strategy at all shock strengths, and performs especially well at higher shock strengths. In these simulations, we gauge the level of synchronization by keeping track of the proportion of active cells in the tissue, and show this value over multiple trials of each strategy when the excitatory pulse strength is 2.89 V/cm in Fig 15. We note that we use a different metric than we did earlier because it is a more natural measurement of the synchronization of the cells, and, unlike in the Karma model, the pattern of hyperpolarization and depolarization caused by the virtual electrodes does not dominate this measurement. Over multiple trials, after the final pulse of the optimal strategy has been applied, an average of 96.8 percent of cells are active, compared to 90.3 and 91.8 percent for the 80 ms and 40 ms pulsed strategies. This increased proportion of activated cells for the optimal stimulus translates into a higher probability that spiral waves will not be able to sustain reentry.

**Fig 16:**
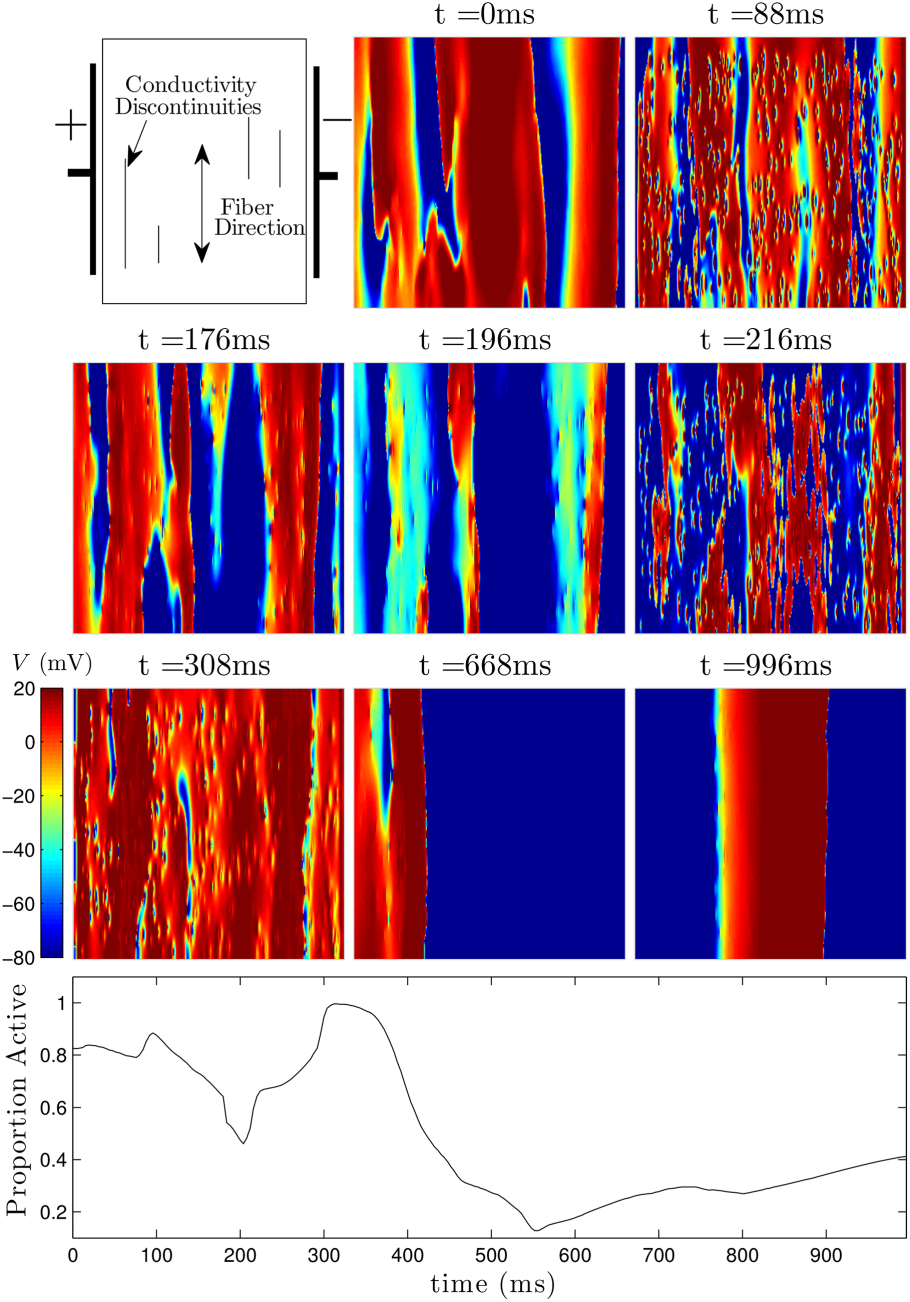
A representative trial using the optimal strategy with excitatory shocks given with 2.89 V/cm. The pulsing strategy manipulates the tissue so that soon after the final pulse at *t* = 308ms, most of the cells are excited, and spiral waves cannot continue to propagate through the medium. The bottom panel shows the proportion of active cells as a function of time. The colorbar presented here applies to all simulations using the ten Tusscher-Panfilov model.

**Fig 17:**
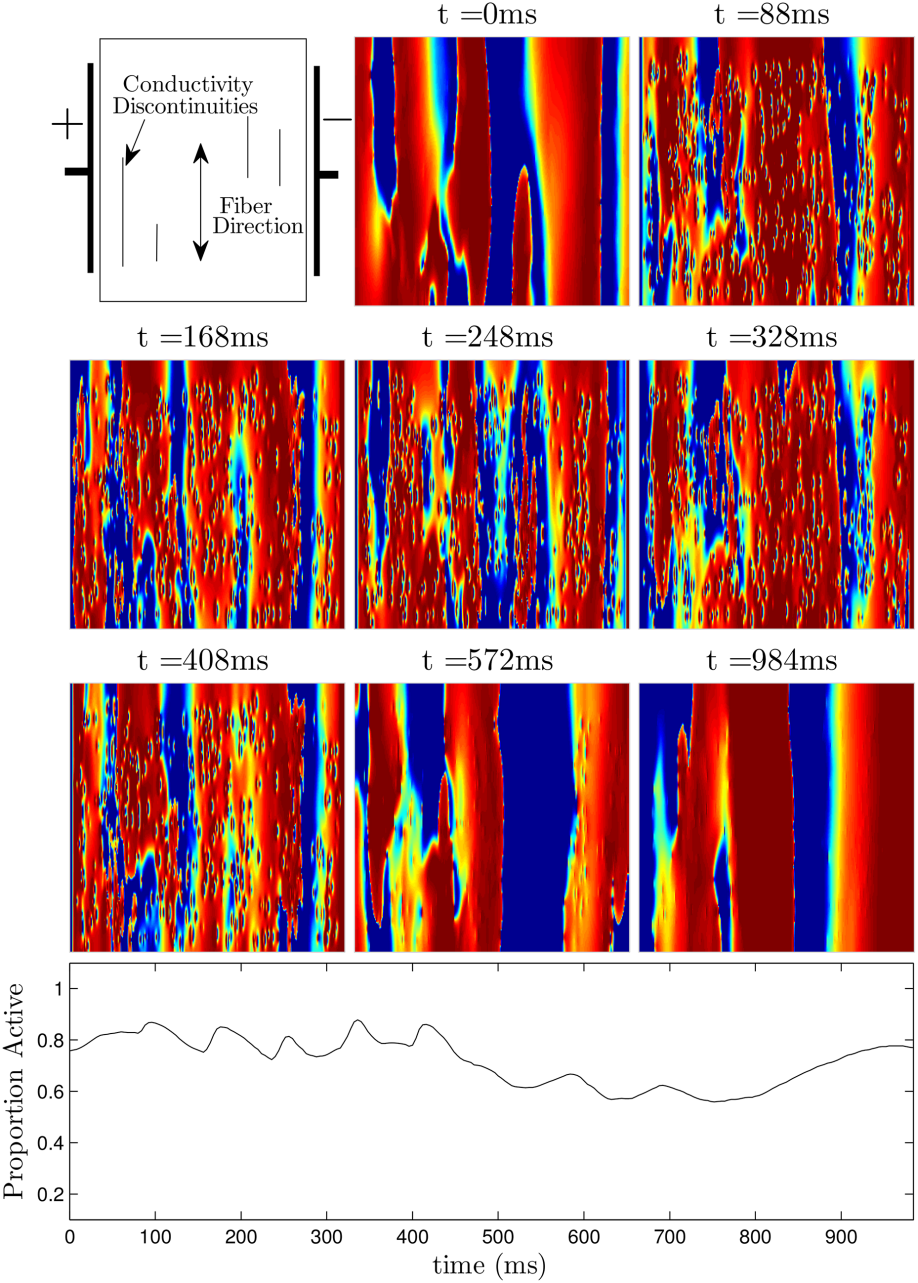
A representative trial using the 80 ms pulsed strategy with shocks at 2.89 V/cm. Soon after the final pulse at *t* = 408ms, large portions of tissue are still quiescent, allowing spiral waves to persist. The bottom panel shows the proportion of active cells as a function of time.

A representative trial using the optimal strategy with excitatory input at 2.89 V/cm is shown in Fig 16 (See also S7 File). At *t* = 84ms, the first excitatory shock excites some of the quiescent tissue. At *t* ≈ 184 ms, the strictly hyperpolarizing pulse is applied, and tissue near the wave front is not affected, while tissue which is closer to becoming refractory recovers faster. The final two excitatory shocks are able to excite nearly all of the remaining tissue, so that the spiral waves are eliminated. Note that the colorbar in Fig 16 applies to all simulations of the ten Tusscher-Panfilov model. A representative trial using the 80 ms pulsed strategy at 2.89 V/cm is shown in Fig 17 (see also S8 File). Notice that in contrast to the simulation shown in Fig 16, after the last excitatory pulse at *t* = 408 ms, large portions of tissue remain quiescent, and spiral waves persist in the medium.

## Discussion and Conclusion

We have proposed a methodology which can be used to find an efficacious series of pulsed inputs to synchronize the activity of myocardial tissue, which increases the likelihood of preventing spiral wave reentry. In a simple two-dimensional model, we were able to design a series of low-voltage excitatory shocks which eliminated reentrant spiral waves with a much higher success rate than a single, high-voltage shock. Furthermore, the resulting optimal pulse sequence was at least as good as a fixed rate pulsing strategy which needed to be found through trial and error (see panel (E) of Fig 4). Using the more realistic ten Tusscher-Panfilov model, we were also able to design a series of excitatory and inhibitory pulses which greatly synchronized the tissue, and led to a high success rate when compared to other single- or multi-pulse strategies. These results suggest that fixed time pulsatile stimulation as currently implemented by low energy strategies may be far from optimal and that the development of strategies to synchronize cardiac cell activity could offer significant room for improvement.

These results represent a proof of concept that the proposed dynamic programming methodology could be used to determine a series of perturbations to cardiac tissue which promote synchronization and eliminate spiral waves, which are thought to be responsible for fibrillation. This dynamic programming methodology could be feasibly implemented experimentally, because it only requires knowledge of how a particular stimulus affects individual cells at certain phases, which can be found by measuring cell action potential durations using, for example, microelectrode recordings of single cell activity or with optical mapping techniques [55] [56]. Furthermore, including the possibility of more than one type of stimulus can greatly improve the dynamic programming algorithm’s ability to find a series of pulses to synchronize the activity of the myocardial tissue. In this work we have only considered the possibility of excitatory and strictly hyperpolarizing perturbations to the cardiac tissue, but other stimuli could easily be considered by the algorithm, provided that the effect on the cells can be experimentally determined.

With regard to optimality of the resulting pulsing patterns, the optimization process is dependent on the ability to accurately measure restitution curves from a given system, a task which is not trivial in an experimental system. In living cardiac tissue, restitution curves are known to vary over time due to pacing history [57, 58]. Furthermore, spatially varying intracellular ion concentrations can result in local variations in the dynamics and a single pulsing pattern may not be optimal in different locations. In this work, we have not fully addressed how errors in the measured restitution curves affect the optimality of the resulting pulsing pattern. Despite these limitations, post-hoc analysis of the resulting optimal stimuli in the computational models can reveal potentially unexpected qualities of useful stimuli for defibrillation in a particular model. For instance, for the Karma model, the optimal stimulus contains two relatively long stimuli followed by a more rapid burst of sequential stimuli. For this model, there is no delay between the time the cell repolarizes and can fire an action potential so that the final burst of stimuli synchronize the cells by trapping their states in a region close to the point that the cells repolarize. This means of synchronization does not exist for the ten Tusscher-Panfilov model, because the cells must be repolarized for a sufficiently long time before they can fire a new action potential. In this case, the dynamic programming algorithm reveals that there is no efficient way to synchronize the cells using only excitatory stimuli. When we include the possibility of a strictly hyperpolarizing stimulus, the dynamic programming algorithm finds that an appropriately timed strictly hyperpolarizing stimulus can prematurely repolarize a large portion of the cells so that successive excitatory stimuli can synchronize the remaining cells (see Fig 12).

While the results suggest a promising new strategy, they are not without limitations. For instance, we did not test the defibrillation strategy in the presence of larger discontinuities, which may be present in some hearts and would allow spiral waves to be “pinned” in place [59–61]. Furthermore, numerical simulations were performed on a square domain, and did not take the complicated geometry of a real heart into consideration. Also, in this work, we assume that the underlying cell dynamics are homogeneous. If there are different cell types that need to be synchronized, the dynamic programming algorithm could still be implemented by including separate groups with different properties at the expense of increasing the size of the state space X. These issues might need to be considered before performing *in vivo* experiments.

The pulsatile defibrillation strategy proposed in this work was primarily implemented with monophasic shocks with current flowing in a single direction. In a clinical setting, however, defibrillators typically use a biphasic waveform where the second phase of the shock is meant to reverse the flow of current from the first shock. These waveforms have been shown to require significantly less energy to defibrillate than using monophasic shocks [48, 49]. While a full analysis of biphasic defibrillation strategies is beyond the scope of this article, pilot simulations of the Karma model suggest that biphasic defibrillation might increase the efficacy of the defibrillating sequence. Using biphasic excitatory stimuli in pilot simulations of the ten Tusscher-Panfilov model did not significantly alter the success rate of defibrillation. Future work could investigate the problem of optimizing defibrillation waveforms [10, 62], for use with pulsatile defibrillation strategies.

These results suggest that stimuli which achieve greater spatial synchronization of myocardial activity can greatly increase the success rate of defibrillation, and may suggest strategies for optimizing newer antifibrillation pacing strategies [14–17]. When used in conjunction with other stimuli than an excitatory stimulus from an ICD, the synchronizing strategy suggested in this work could reduce the energy required for successful defibrillation and could potentially mitigate the physiological and psychological side effects associated with frequent defibrillation.

## Supporting Information

S1 File In this bidomain simulation of the Karma model, a single pulse fails to eliminate spiral waves in the medium. (MP4)

S2 File In this bidomain simulation of the Karma model, the optimal series of 5 excitatory pulses eliminates spiral waves in the medium. (MP4)

S3 File In this bidomain simulation of the Karma model, the series of 5 excitatory pulses at constant intervals fails to eliminate spiral waves in the medium. (MP4)

S4 File The optimal control strategy determined from the dynamic programming strategy is applied to square patches of tissue from the TNNP model. Each patch is initially equally spaced in *ψ*. The pulsing strategy is effective at synchronizing the activity of the patches.
(MP4)

S5 File Excitatory pulses are applied to 18 square patches of tissue from the TNNP model. Each patch is initially equally spaced in *ψ*. The pulsing strategy does not work for synchronizing the activity of the patches.
(MP4)

S6 File In this bidomain simulation of the TNNP model, the single excitatory pulse fails to eliminate spiral waves in the medium. (MP4)

S7 File In this bidomain simulation of the TNNP model, the optimal pulsing strategy eliminates spiral waves in the medium. (MP4)

S8 File In this bidomain simulation of the TNNP model, the series of 5 excitatory pulses at constant intervals fails to eliminate spiral waves in the medium. (MP4)
